# Mors Gaudet Succurrere Vitae. The Role of Clinical Autopsy in Preventing Litigation Related to the Management of Liver and Digestive Disorders

**DOI:** 10.3390/diagnostics11081436

**Published:** 2021-08-09

**Authors:** Stefano D’Errico, Martina Zanon, Michela Peruch, Monica Concato, Martina Padovano, Alessandro Santurro, Matteo Scopetti, Vittorio Fineschi

**Affiliations:** 1 Department of Medicine, Surgery, and Health, University of Trieste, Strada di Fiume 44, 34149 Trieste, Italy; sderrico@units.it (S.D.); martina.zanon@virgilio.it (M.Z.); michela.peruch95@gmail.com (M.P.); monica.concato@studenti.units.it (M.C.); 2 Department of Anatomical, Histological, Forensic and Orthopaedic Sciences, Sapienza University of Rome, Viale Regina Elena 336, 00185 Rome, Italy; martina.padovano@uniroma1.it (M.P.); alessandro.santurro@uniroma1.it (A.S.); vittorio.fineschi@uniroma1.it (V.F.)

**Keywords:** hospital autopsy, digestive and liver diseases, error prevention, risk management, adverse events, gastroenterology claims, patient safety, healthcare quality

## Abstract

Over the last 50 years, the number of clinical autopsies has decreased, but their role in assessing cause of death and clinical performance is still acknowledged. Few publications have studied their role in malpractice claim prevention. The paper aims to highlight the role of clinical autopsy in preventing errors and improve healthcare quality. A retrospective study was conducted on 28 clinical autopsies performed between 2015 and 2021 on patients dead unexpectedly after procedures for the diagnosis and treatment of digestive and hepatic diseases. After an accurate analysis of medical records and consultation with healthcare professionals, all cases were subjected to autopsy and histopathology. The data obtained were analyzed and shared with the risk-management team to identify pitfalls and preventive strategies. Post-mortem evaluations confirmed the clinical diagnosis only in six cases (21.4%). Discordances were observed in 10 cases (35.7%). In the remaining 12 cases (42.9%) the clinical diagnosis was labeled as “unknown” and post-mortem examinations made it possible to document the cause of death. Post-mortem examinations can concretely enrich hospital prevention systems and improve patient safety. The methodological approach outlined certainly demonstrates that, even in the risk-management field, “mors gaudet succurrere vitae” (“death delights in helping life”).

## 1. Introduction

The management of adverse events in medicine is a major challenge for different health systems around the world. Over the years, the approach to clinical risk has seen the use of different reactive and proactive methods. In this regard, hospital legal medicine unquestionably constitutes a privileged observatory in relation to the dual activity of clinical autopsy and litigation management. In fact, despite being historically involved in the management of claims, legal medicine is fully involved with clinical risk management and patient safety and carries out hospital autopsies to improve a policy of transparency and as a tool for measuring the quality of performance.

Regarding the autopsy, the assessment represents the gold standard for establishing the cause of death and constitutes a crucial aid for evaluating the management and performance of the healthcare structure; in fact, many studies emphasize its role in the continuous evolution of medical science [1,2], both in the forensic field and clinical setting. Post-mortem investigations in the clinical setting also allow the accurate reconstruction of care pathways to determine the repercussions of health treatments on the patient due to the possibility of integrating the objective findings with the evidence inferable from the analysis of the health documentation and from the direct comparison with the professionals [3,4,5,6]. The autopsy permits the ensuring of transparency through the active involvement of wards and families [7]. In fact, in the case of suspected medical professional liability, the autopsy enables useful objectivity for the evaluation of medical conduct and for ascertaining the reality of the alleged facts; such an assumption clarifies the considerable implications in the dynamics of litigation prevention and reduction of the costs of medical liability [8].

The phenomenon of defensive medicine currently represents a significant element in the decrease in hospital autopsies [9,10]. In particular, the fear of possible litigation entails the renunciation of a fundamental diagnostic tool for the clarification of clinical questions and related to the effects of some pathologies on the individual [11,12].

The reconstruction of the interventional modalities and the identification of the precise pathophysiological mechanisms underlying the death allow orienting the management and care policy of the facilities. Autopsy findings represent a fundamental tool for risk mapping, root cause analysis, and identification of adverse events. Although often considered an unnecessary assessment in relation to costs, autopsy therefore plays a key role in the quality and cost containment of healthcare [13].

As regards claims management, the specialties most involved in litigation are represented by the surgical branches with preponderance of gynecology and general surgery [14,15]. In the United States, over 15% of practicing general surgeons face malpractice claims; according to further studies, almost all general surgeons over the age of 65 faced at least one complaint of professional malpractice during his or her career [16]; furthermore, it has been estimated that in the United States a physician spends an average of 50.7 months over a 40-year career in claims that in more than 70% of cases do not involve any sentence or payment [17]. As for gastroenterology, the rate of claims paid per 1000 physicians each year has declined over the past two decades; but the average payment amounts showed an increase. Claims paid refer mainly to harmful events caused by presumed diagnostic or therapeutic (mainly surgical) errors. Most of the damage resulting from inadequate gastroenterological behaviors is serious and even includes death. About 14% of gastroenterologists face medical liability claims each year, but only 2% of claims are actually paid. In detail, 78.3% of the doctors involved in the claims are between the ages of 35 and 54 and 66% of the total have a previous history of claims. The main reasons behind the claims are represented by the misdiagnosis and the inadequate execution of a procedure; with specific reference to this last aspect, 52% of the claims concern procedures on the large intestine, 16% procedures on the gallbladder and biliary tract, and 11% procedures on the esophagus [18]. Data collected by the Physician Insurers Association of America states that gastroenterological disputes represent only 1.8% of all claims; 25% of these ended with a verdict or agreement between doctor and patient, 70% with a waiver or rejection, while the remaining 5% with a favorable outcome for the doctor [19,20]. In Italy, data relating to health disputes at the national level are not available, but in the last decade, the rate of claims has grown in parallel with the increase in the average amount paid per claim reported [21]. Regardless of the wide variability between the international legislative frameworks, the autopsy evaluation represents an undisputed tool for preventing litigation, so much so that it deserves to be routinely included in the diagnostic path of deaths not clinically defined to guarantee objectivity and transparency.

So far, only a few studies have framed the autopsy as a possible useful tool in reactive risk management [22,23,24]. Given the topicality and significance of the problem, the present study aims to demonstrate the importance of clinical autopsy in deaths following imaging, endoscopic, or surgical procedures for the diagnosis and treatment of digestive and hepatic diseases. In particular, the comparison of post-mortem findings with clinical evidence will emphasize the role of clinical autopsy in identifying the suboptimal steps of care paths as well as driving the policies on quality and patient safety.

## 2. Materials and Methods

A retrospective study was conducted on 28 clinical autopsies performed between 2015 and 2021. The inclusion criteria were: (1) sudden unexpected deaths; (2) recent (less than 30 days) imaging, endoscopic, or surgical procedures; and (3) clinical diagnosis of digestive and hepatic disease; the term of 30 days has been identified by convention as the surgical outcomes can be considered stabilized in this time frame. The exclusion criteria were: (1) autopsy ordered by the prosecutor (judicial autopsy); and (2) older (over 30 days) imaging, endoscopic, or surgical procedures. During the enrollment phase, no limits were set regarding age, length of stay, and type of procedure performed.

Preliminarily to each autopsy, accurate analysis of the medical records was carried out; besides, there was a consultation with the health professionals implicated in the care of the deceased. All the examinations were executed in the presence of medical consultants from the ward and the family of the deceased—as well as the pathologist.

Consistent with current protocols [25,26,27,28], the autopsy was performed with an external and internal examination. For each autopsy, a targeted technical approach was identified—oriented based on the patient’s clinical features and the formulated diagnostic suspicion—to determine the natural cause of death or the relationship with inadequate healthcare. The external examination was aimed at investigating the possible presence and condition of the surgical site as well as detecting additional signs of the primary disease and the medical care (e.g., signs of acupuncture); moreover, it made it possible to detect the aids used during the healthcare path (such as central venous catheters, respiratory supports, and bladder catheters). During the internal examination, all body cavities were investigated; particular attention was devoted to studying the body districts involved in diagnostic and therapeutic procedures. All examinations were completed by taking samples of organs and tissues for subsequent microscopic investigations.

The carrying out of histopathological examinations involved the microscopic observation of preparations stained with the hematoxylin–eosin technique starting from samples taken during autopsy suitably formalin-fixed and paraffin-embedded (FFPE).

The obtained data were analyzed, compared with clinical diagnoses, and shared with the risk-management team to identify pitfalls and preventive strategies.

Considering the sample size, the descriptive intent of the study, and the impossibility of identifying comparable groups for statistical purposes, a descriptive statistical analysis was carried out with the calculation of the frequencies in absolute and relative terms.

## 3. Results

The study included a sample of 28 cases of patients who died unexpectedly following procedures for the diagnosis and treatment of digestive and liver diseases between 2015 and 2021 (Table 1).

Out of the 28 cases, 15 (53.6%) were men and 13 (46.4%) women. The mean age was 71.9 years (range from 44 to 93 years).

**Figure 1:**
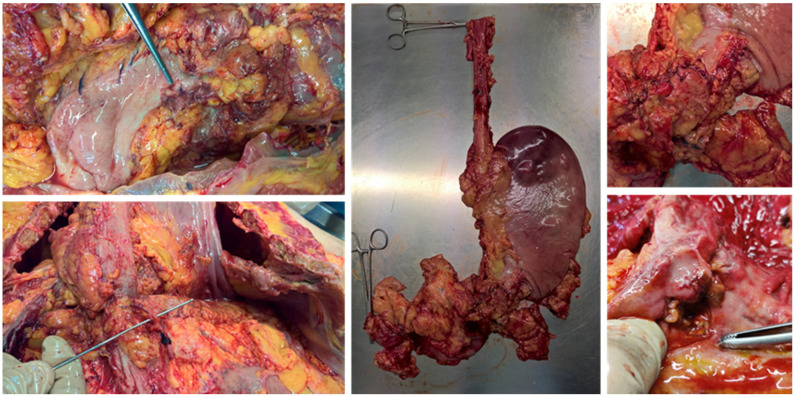
Autopsy investigation on case no. 17. Surgical treatment of bleeding duodenal ulcer and erosion of the superior pancreaticoduodenal artery. Evidence of correct surgical hemostasis.

Regarding the procedures performed, imaging methods were the most frequently involved (eleven; 39.2%); in detail, abdominal ultrasonography was performed in seven cases (25%), X-rays in two cases (7.1%), and CT and FAST scan in the remaining two cases (7.1%). Endoscopic procedures were executed in seven cases (25%) and included esophagogastroduodenoscopy (EGD) (four; 14.2%) and endoscopic retrograde cholangiopancreatography (ERCP) (three; 10.8%). Surgical procedures were performed in a further seven cases (25%), including laparoscopy (one; 3.6%) and laparotomy (six; 21.4%) (Figure 1). The remaining three cases (10.8%) included in the study underwent post-transplant liver biopsy, trans-arterial embolization (TAE) of the bleeding branches of the right hepatic artery (RHA), and a paracentesis for the cytological evaluation of a peritoneal effusion.

From the analysis of the health documentation as well as from the audit with the physicians involved in the healthcare path, it emerged that the cause of death formulated in the clinical setting remained unknown in 12 cases (43%). In the other 16 cases (57%) the clinical diagnosis of death involved an acute cardiocirculatory disorder (five; 17.8%), a disease of the gastrointestinal system and the liver (six; 21.4%), and a systemic infectious process (five; 17.8%).

In all cases, the post-mortem examination obtained objective evidence of the condition responsible for death. In detail, the pathologic diagnosis of death involved an acute cardiocirculatory disorder (six; 21.4%), a respiratory disorder (five; 17.8%), a disease of the gastrointestinal system and the liver (fourteen; 50%), and a systemic infectious process (three; 10.8%).

**Figure 2:**
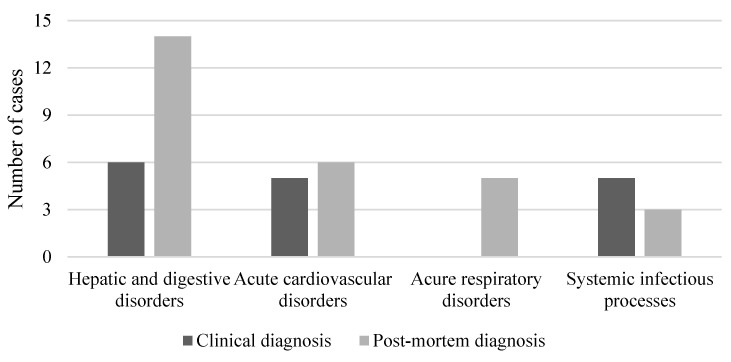
Distribution of cases according to the type of definitive diagnosis.

**Figure 3:**
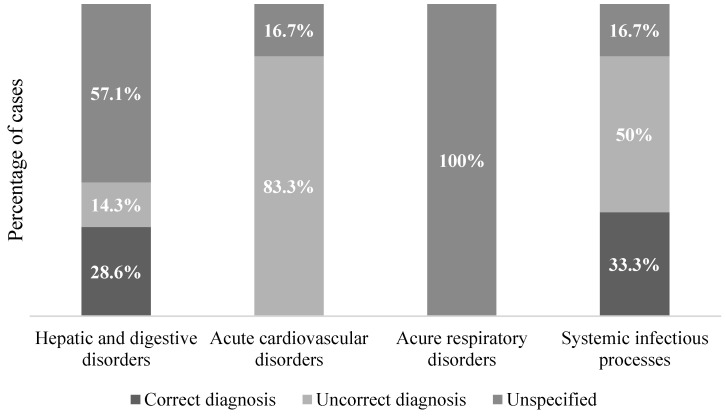
Distribution of cases according to adequacy of clinical diagnosis.

Comparing the evidence obtained through the examinations included in the present study and the clinical diagnoses, it was possible to highlight multiple discrepancies (Figure 2 and Figure 3); in fact, post-mortem evaluations confirmed the clinical diagnosis in six cases (21.4%). In 10 cases (35.6%) the diagnosis was discordant, and post-mortem exams confirmed an alternative cause of death. In cases with unknown clinical diagnosis, autopsy and histopathological exams detected cardiovascular disorders (four; 14.3%), acute respiratory failure (two; 7.1%), hepatic and digestive disorders (five; 17.8%), and a systemic infectious process (one; 3.6%).

**Figure 4:**
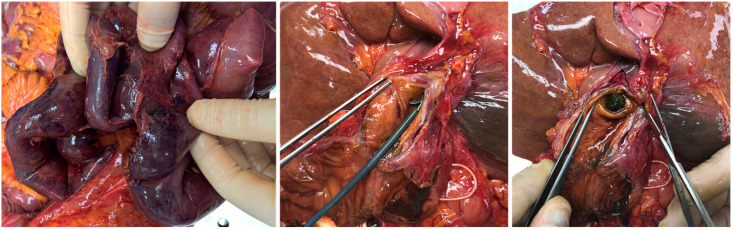
Autopsy investigation on case no. 16. Intestinal ischemia associated with peritonitis. The dissection of the biliary tract showed the correct positioning of an endoprosthesis and a lithiasis of the main biliary tract.

**Figure 5:**
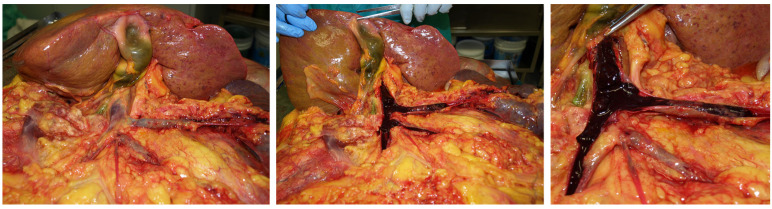
Autopsy investigation on case no. 3. Findings of massive thrombosis of the portal vein.

As regards the diseases underlying death it was possible to detect heterogeneous conditions, such as coronary artery disease (CAD) (two; 7.1%), rupture of abdominal aortic aneurism (AAA) (one; 3.6%), acute heart failure (AHF) (two; 7.1%), myocarditis (one; 3.6%), pulmonary embolism (two; 7.1%), bacterial pneumonia (two; 7.1%), aspiration pneumonia (one; 3.6%), upper gastrointestinal bleeding (four; 14.3%), hepatic bleeding (two; 7.1%), intestinal perforation (two; 7.1%), intestinal occlusion with or without ischemia (four; 14.3%) (Figure 4), portal vein thrombosis (one; 3.6%) (Figure 5), ascites (one; 3.6%), and sepsis (three; 10.8%).

Concerning the relationship between clinical autopsy and litigation in the 28 cases analyzed, a significant ability of the former towards the latter was highlighted. In particular, in 22 cases (78.6%), the participation in the autopsy of the physicians of the ward and the trusted physicians of the relatives of the deceased allowed the sharing of evidence useful for resolving doubts regarding the behavior of the health professionals involved in the care. In the remaining six cases (21.4%), a dispute was initiated despite the carrying out of the autopsy; of these, two have been defined through an economic agreement due to the evident inadequacies of assistance, while four are still undefined due to the continuation of the judicial phase.

## 4. Discussion

Patient safety represents a growing challenge in proportion to the increasing complexity of care pathways. A deep understanding of diagnostic and therapeutic processes is a fundamental prerequisite for improving the quality of care. However, the assessment of the critical phases of providing care presents some asperities correlated to a scarce culture of error. In fact, a significant proportion of healthcare professionals are reluctant to report and learn methods based on adverse events because of shame or fear of compromising their reputation.

In US hospitals, a similar context translates into an increase in medical malpractice costs, which grew by an annual rate of 11.8% from 1975 to 2005. Such an attitude towards the conduct put in place by health professionals determines the doubling of the disparity in the costs faced by physicians compared to those of the facilities; in fact, in 1975 the payments to be made by hospitals were 30% lower than those of physicians, while in 2003 the first were 60% lower than the latter [29,30].

Outlining the economic weight of adverse events in terms of direct and indirect costs, it seems appropriate to underline how profitable risk management through reactive and proactive methods is able to limit the use of resources by healthcare facilities.

Among the different tools useful for risk mapping, the hospital autopsy undoubtedly represents an important diagnostic assessment for reactive analysis. Nevertheless, since the second half of the 20th century, in many countries the autopsy rate has undergone a massive decline; the factors involved are many, e.g., social, cultural, and religious beliefs [31], as well as the conviction of clinicians to be able to independently identify the pathological conditions and the related causes of death. Unfortunately, this drop in the rate of post-mortem examinations is a disadvantage, as there is a lack of valid support for medicine. In fact, the autopsy plays a role in protecting science, hospitals, and relatives of the deceased. Nonetheless, post-mortem assessment, representing the gold standard for defining the cause of death, provides valid support to health facilities and family members [32]. As demonstrated in the present study, a proactive risk-management approach can be implemented after a thorough analysis of the health records and audits with the health professionals.

The conducted study demonstrated the educational role of autopsy in orienting the practice of individual professionals and in providing useful insights for planning the policies of the health facilities. In particular, the autopsy definition of the critical-care issues and root causes facilitates the development of internal procedures for the reduction of unwanted events.

Concerning diagnostic imaging, the results obtained alternately show concordance and discrepancy with the literature data, highlighting interesting aspects relating to the diagnostic classification of patients, especially in emergency contexts. According to the current evidence, given the non-specificity of the symptoms, a timely approach based on the execution of abdominal ultrasound, CT scan, and X-rays in case of suspected bowel obstruction is of primary utility to establish any urgency; despite the dependence on the operator, ultrasound is the first assessment that should be performed to establish the real presence of an obstruction, especially in the case of involvement of the small intestine (sensitivity 90–92.4%; specificity 96–96.7%) [33,34]; a similar diagnostic capacity is comparable to the CT and better than X-rays (sensitivity 75%; specificity 66%) [35]. Conversely, CT scan is more accurate in identifying the cause of the obstruction. The evidence obtained from the present study made it possible to highlight how the exclusive execution of imaging methods did not always reach an exact diagnosis. In the enrolled cases, ultrasound was not decisive in diagnosing obstruction while conventional radiology demonstrated the presence of hydro–air levels when used. Such results essentially depended on operator-related factors and technical limitations in performing the exam. In suspected bowel perforations, ultrasound is indicated for the detection of pneumoperitoneum. The non-use of ultrasound in the present case series is justified by the fact that in one case, given the severity of the clinical conditions, an exploratory laparotomy was performed and in the other, given the positive history of AAA, a CT scan was performed to rule out a rupture; about the latter case, although the CT scan represents the method with the highest sensitivity in detecting intraperitoneal free gas [36,37,38], it was not possible to detect the radiological signs of perforation in relation to the patient’s intra-procedural death. Furthermore, according to the ATLS protocol [39,40,41], the FAST scan was performed in a hemodynamically unstable patient, without however allowing the diagnosis of bleeding from esophageal varices.

Gastrointestinal bleeding is a common reason for emergency room access that frequently presents with hematochezia, melaena, or hematemesis; such presentation can lead to a condition of clinical instability which requires appropriate management [42,43]. In the suspicion of gastrointestinal hemorrhage, in the presence of stable clinical conditions, it is advisable to perform an endoscopic procedure for diagnostic and therapeutic purposes; this is the case of one of the study subjects who, despite the sudden cardiovascular collapse prevented the completion of the examination, was correctly initiated to the EGD in consideration of the positivity of the history for hematemesis and the stability of the clinical conditions. On the other hand, in cases of clinical instability, the criticality and non-specificity of the presenting symptoms have precluded the possibility of performing endoscopic procedures, allowing only to conduct imaging tests of limited diagnostic capacity in consideration of the intrinsic limitations of the methods.

In reference to the accuracy of the clinical diagnosis with respect to the autopsy diagnosis, the high percentage of incongruous or unknown diagnoses (22; 78.6%) can be explained by the care context. In fact, most of the examined cases were characterized by hospitalizations in emergency operating units and critical conditions upon admission such as not to allow prompt classification. In other cases, the diagnostic process was limited by the inability to collect detailed anamnestic information. Again, in some cases (two; 7.1%) the diagnostic process was interrupted by the patient’s intraprocedural death. Focusing the discussion on cases of discrepancy between clinical and autoptic diagnosis, the highest diagnostic accuracy has been achieved in the field of gastrointestinal and hepatic diseases. On the other hand, excessive recourse to the diagnosis of cardiac death was found with a significant number of cases in which the cause was attributable to another system; moreover, in one case, autopsy made it possible to demonstrate the existence of a disease other than that hypothesized by the clinicians, despite the correct attribution to the cardiovascular system. A further relevant aspect was the finding of a low index of clinical suspicion for infectious and embolic diseases of the respiratory system. The data relating to the diagnostic pitfalls in the field of cardiovascular and respiratory diseases show absolute consistency with the evidence of the literature relating to the conspicuous amount of litigation deriving from similar events [44,45,46].

The experience gained in approaching the cases described has unquestionably demonstrated the value of transparency. The participation in the autopsy activities of all the parties involved allowed, in most cases, the effective reconstruction of the care process. Of particular interest is the experience of two procedures for surgical hemostasis of bleeding peptic ulcers in which inadequate surgical conduct was suspected following death; in these cases, the autopsy exploration of the surgical site demonstrated the tightness of the sutures, dispelling any hypothesis of inadequacy.

From the point of view of clinical risk, in addition to the audits carried out before each autopsy, reactive risk-management measures were implemented, such as the improvement of protocols for the diagnosis of intestinal obstructions. Currently, evidence-based proactive management measures are being evaluated.

Conclusively, the obtained results demonstrate the usefulness of the autopsy as a tool for preventing litigation through transparency and the prerogative of ascertaining the reality of the events. In particular, the objective vision provided by the port-mortem examinations makes it possible to dispel any suspicions regarding the inadequacies of healthcare. Likewise, post-mortem assessments have been shown to profitably contribute to risk management by identifying suboptimal steps in care pathways and allowing immediate analysis of the underlying causes of adverse events. Investment in hospital post-mortem diagnostics can therefore concretely enrich prevention systems and improve the safety of care. Therefore, it is appropriate to state that even in the field of risk management, “mors gaudet succurrere vitae” (“death delights in helping life”).

## Figures and Tables

**Table 1:** Summary of the 28 cases included in the study.

Case	Gender	Age	Admission Diagnosis	Procedure	Discharge Diagnosis	Autopsy Diagnosis
1	M	69	Abdominal pain, hepatic cirrhosis	Abdominal ultrasonography	Unknown	AAA rupture
2	M	78	Liver transplant	Biopsy	Unknown	CAD, SCD
3	M	61	Jaundice, fever	Abdominal ultrasonography	Sepsis	Thrombosis of the portal vein
4	M	67	Shock in hepatic cirrhosis	Abdominal ultrasonography	Unknown	Bleeding peptic ulcer
5	F	71	Colon cancer	Right hemicolectomy	Sepsis	Sepsis, MODS
6	M	70	Alcoholic liver disease	Abdominal ultrasonography	Unknown	Sepsis
7	F	80	Decompensated liver cirrhosis	EGD	Unknown	Bleeding cirrhotic nodule
8	M	55	Jaundice, hepatitis C	EGD	Broncho-pneumonia, sepsis	Pulmonary embolism
9	F	71	Crohn’s disease	Right hemicolectomy	Intestinal ischemia	Aspiration pneumonia
10	M	77	Bleeding gastric ulcer	Surgical hemostasis	Unknown	Acute heart failure
11	F	67	Acute abdomen	Abdominal ultrasonography	Unknown	Bowel obstruction
12	F	83	Diarrhea	Abdominal ultrasonography	Unknown	Bowel obstruction
13	F	87	Abdominal pain, hepatitis C	EGD	Unknown	Pulmonary embolism
14	M	80	Cholecystitis	Laparoscopic cholecystectomy	Unknown	CAD, SCD
15	F	86	Stones of the main biliary tract	ERCP	Sepsis	Sepsis due to biliary fistula
16	M	82	Cholecystitis, cholangitis	ERCP	Lithiasic cholangitis, sepsis	Intestinal ischemia with peritonitis
17	M	84	NSAIDs duodenal ulcer	Surgical hemostasis	Bleeding duodenal ulcer	Acute heart failure
18	F	69	Duodenal perforation	Laparotomy for duodenal suture	Unknown	Bacterial pneumonia
19	F	84	Bowel obstruction	Abdominal X-ray	Bowel obstruction	Bowel obstruction and ischemia
20	F	64	Bleeding from branches of the RHA	TAE	Bleeding hepatic cancer	Hemoperitoneum from bleeding HCC
21	M	57	Jaundice	ERCP	Cardiocirculatory collapse	Bacterial pneumonia
22	M	52	Intestinal perforation	Exploratory laparotomy	Stercoraceous peritonitis	Intestinal obstruction, perforation
23	F	57	Ascites in liver cirrhosis	Paracentesis	Liver cirrhosis in ascitic phase	Ascites
24	M	81	Hematemesis	EGD	Cardiovascular collapse during EGD	Bleeding duodenal ulcer
25	F	44	Shock in gastroenteritis	Abdominal ultrasonography	Cardiocirculatory collapse	Myocarditis
26	M	69	Abdominal pain in patient with AAA	Abdominal CT	Cardiocirculatory collapse during CT	Perforated duodenal ulcer, peritonitis
27	M	93	Shock	FAST scan	Unknown	Bleeding esophageal varices
28	F	74	Persistent vomiting, shock	Abdominal X-ray	Cardiocirculatory collapse	Bleeding duodenal ulcer

AAA, abdominal aortic aneurism; CAD, coronary artery disease; SCD, sudden cardiac death; MODS, multiorgan dysfunction syndrome; EGD, esophagogastroduodenoscopy; ERCP, endoscopic retrograde cholangiopancreatography; RHA, right hepatic artery; TAE, transcatheter arterial embolization; HCC, hepatocellular carcinoma; ARF, acute renal failure; and IBD, inflammatory bowel disease.

## Data Availability

The data used to support the findings of this study are available from the corresponding author upon request.
